# An Unusual Case of Sudden Collapse in the Immediate Postoperative Period in a Young Healthy Female with Myxofibroma of the Maxilla

**DOI:** 10.1155/2013/596758

**Published:** 2013-07-30

**Authors:** Manila Singh, Saket Singh

**Affiliations:** ^1^Department of Anesthesia and Intensive Care, G.B. Pant Hospital, E-269, East of Kailash, New Delhi, India; ^2^Department of C.V.T.S, G.B. Pant Hospital, New Delhi, India

## Abstract

Benign myxofibromas of heart are well known to cause systemic inflammatory mediator release causing multiple complications ranging from fever and widespread effusions to DIC and shock. We report that in a particular case of maxillary myxofibroma, a shock-like state and widespread serous cavities effusion presented in the immediate postoperative period. The occurrence was possibly due to release of inflammatory mediators by the tumour, disseminated during tumour resection causing diffuse capillary leak, precipitated by fluid resuscitation, leading to decrease in plasma oncotic pressure.

## 1. Introduction

Odontogenic fibromyxoma is a rare benign tumour arising from odontogenic mesenchyme [1]. Shared field for airway and surgical access [2] is the main anaesthetic challenge along with a smooth postoperative course necessary for maintaining a patent airway as well as the suture line. Any systemic complications related to inflammatory mediator release by the tumour have not been reported before as the available data suggests.

The written informed consent for publication of data was taken postoperatively from the patient and her parents when her condition stabilized on postoperative day two.

## 2. Case Report

A 13-year-old otherwise healthy female was scheduled to be operated on for myxofibroma of maxilla by a combined team of neurosurgeons and dental surgeons. On routine preanaesthetic check-up, the patient was perceived to be a healthy, 40 kg female, not yet having attained menarche. The patient was receiving dexamethasone 4 mg 12-hourly preoperatively. Routine preoperative biochemical and radiological investigations comprising complete blood count (CBC), LFTs (liver function tests), KFTs (kidney function tests), CXR (chest X-ray), ECG, and PT/INR were within normal limits.

After application of routine monitors, anaesthesia was induced with fentanyl 2 mcg/kg, midazolam 2 mg, and propofol 80 mg. Nasal intubation was facilitated by rocuronium 35 mg and McGill's forceps. Maintenance of anaesthesia was done with inhalational isoflurane and oxygen nitrous mixture with supplementation of relaxant guided by half-hourly TOF count which was kept below two. Hemimaxillectomy using a Weber Fergusson incision with tumour excision was done. Patient was reversed and extubated after a sustained head lift of >5 seconds, and a TOF ratio of 0.9 was demonstrated.

Patient was shifted to the neurosurgical ICU in the immediate postoperative period for overnight monitoring of vitals. Blood gases immediately after shifting and 1 hr thereafter were within normal limits. Pantoprazole infusion was started in the ICU in view of the history of steroid intake preoperatively and continuation of the same postoperatively.

2.5 hrs after arrival, her blood pressure suddenly dropped down from 108/60 mmhg to 66/45 mmhg, and the patient became unconscious. Heart rate was maintained between 110 and 108 per min throughout. Oxygen saturation decreased from 96% to 85%.

RL 1000 ml followed by 500 ml of Haes-steril 6% was administered over 20 minutes to raise the CVP from 6 mmhg to 10–12 mmhg. Patient was ventilated with bag and mask while the intubation tray was kept ready. She became conscious within 5 minutes, and the BP gradually increased to 90/50 mmhg. Decision for intubation, which had the potential to disrupt the suture line as well as being a difficult one owing to the absence of right maxilla and hard palate, was withheld.

Monitoring was continued. Oropharyngeal suction was done, and gauze packs used to pack the maxilla were examined (for any increased bleeding) to have the expected amount of soakage. 50 ml of altered blood was aspirated from Ryle's tube which did not explain the magnitude of hypotension. 

After gaining consciousness, the patient complained of diffuse pain per abdomen. On examination, generalised guarding was present per abdomen. Haemoglobin of the patient was 8 gm% after volume infusion, and the patient was transfused 1 unit of packed RBCs. Five mg/kg/min of dopamine infusion was started to support the borderline BP of 86–90 mmhg systolic which increased to >100 mmhg. CVP continued to be 10-11 mmhg after volume infusion. after resuscitation, the SpO_2_ increased to 89% without O_2_ supplementation and was maintained at 90–94% with O_2_ supplementation.

**Figure 1:**
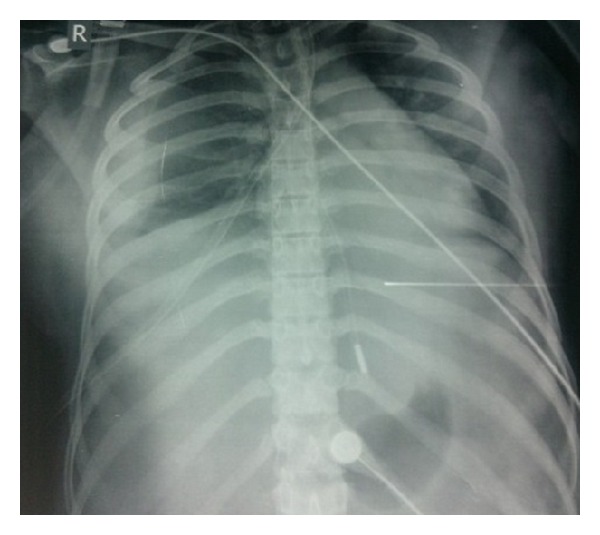
Moderate pleural effusion with a large cardiac silhouette suggestive of pericardial effusion.

**Figure 2:**
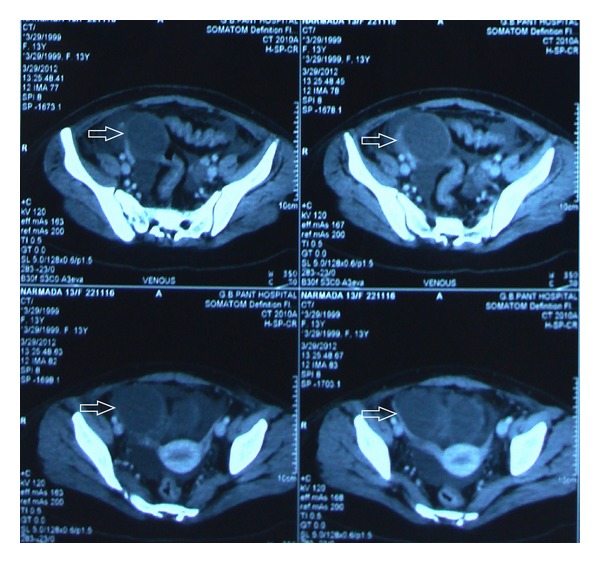
CT sections showing a hemorrhagic ovarian cyst.

A chest X-ray was ordered next which showed signs of pleural effusion (Figure 1). Ultrasound and CT whole abdomen revealed gross ascites and a hemorrhagic ovarian cyst (4.5 cm × 5 cm) (Figure 2), and echocardiography revealed pericardial effusion. Blood investigations revealed hypoalbuminemia (serum albumin −2.5 mg/dl). Haemoglobin increased to 10 gm% after transfusion. Ascitic fluid biochemistry revealed a SAAG of 1 indicating absence of a cardiac, gastrointestinal, or renal cause of ascites.

Over the next 24 hrs, PaO_2_ of the patient dropped down from 54 mmhg to 46 mmhg. Dopamine was increased to 8 *μ*/kg/min to maintain a systolic blood pressure over 90 mmhg. Noninvasive BiPAP of 15 and 7 mmhg was started to which the patient responded well, and the SpO_2_ increased to 95% while the PaO_2_ increased to 57 mmhg over the next 1 hr.

**Figure 3:**
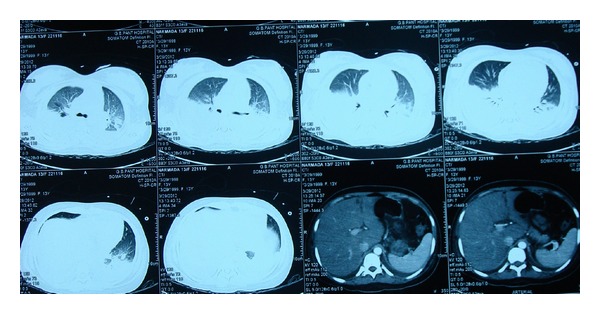
CT sections showing bilateral pleural effusion with atelectatic lower zones.

**Figure 4:**
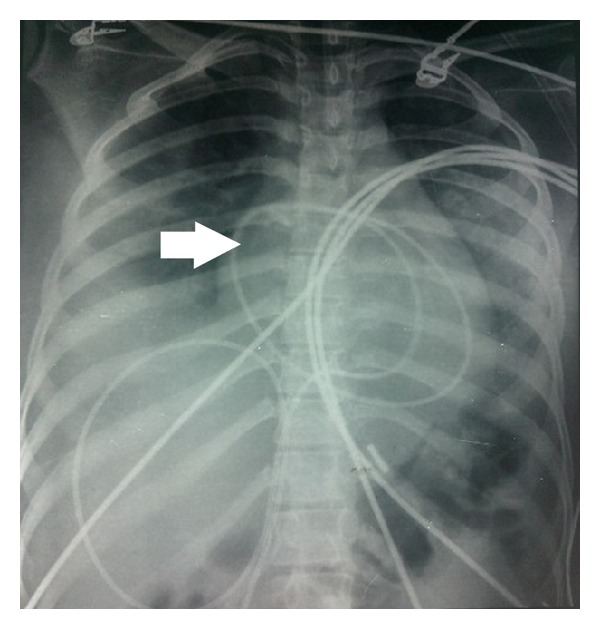
CXR film showing pigtail catheter in situ (arrow).

Contrast enhanced computed tomography (CECT) and repeat echocardiography were done which revealed a cardiac tamponade due to moderate pericardial effusion and bilateral pleural effusion with collapse of lower lobes and large volume ascites (Figure 3). Ascites was drained under ultrasound guidance, and over 4 litres of fluid was drained over 2 days in 6 divided sessions. Dopamine infusion had to be increased to 9 *μ*/kg to maintain the BP over 90/50 mmhg. A pigtail catheter was inserted, and 150 ml of pericardial fluid was drained immediately (Figure 4). USG guided pleural tapping was also done, and up to 300 ml of fluid was removed from each side. Ascitic and pleural fluids were negative for AFB. 

By the next day, dopamine was tapered and stopped. Pleural and pericardial fluid adenine deaminase (ADA) was of a borderline value of 35 IU excluding tuberculosis as the cause of such widespread serous cavities effusion. 

Based on serum-ascites albumin gradient (SAAG) value and USG findings, serum *α* fetoprotein, C-reactive protein (CRP), *β* human chorionic gonadotropin (HCG) and cancer antigen-125 (CA-125) were done as advised by the gynaecology team. CA125 was found to be markedly raised, with an absolute value of 241 U/ml (normal < 35 IU/ml) [3]. CRP was 38 mg/L. Serum alfa fetoprotein (AFP) and *β* HCG were within normal limits.

A suspicion of a gynaecological malignancy was made, and it was decided to transfer the patient to gynaecology unit for management after recovery.

**Figure 5:**
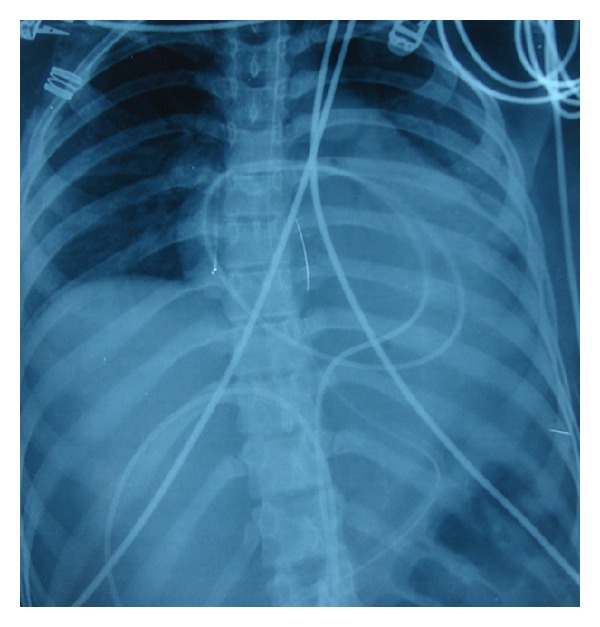
Repeat CXR film showing no increase in pleural or pericardial effusions.

Over the next 2 days, blood gases improved remarkably, and the patient was gradually weaned off NIV. Repeat CXR (Figure 5) showed no signs of reaccumulation of pleural or pericardial effusion. MRI of abdomen and pelvis showed no significant finding. Abdomen was soft, and no pain per abdomen or guarding was present. Oxygen supplementation was stopped over the next 4-5 hrs. PaO_2_ on room air was 70 mmhg. All other blood investigations were within normal limits, and serum albumin levels had normalised. Over the next week, CA-125 levels decreased to 32 U/ml and the size of the cyst on repeat pelvic ultrasound 8 days later showed a decrease in size to 3.5 cm. CRP levels also decreased to 15 mg/L. Interleukin-6 (IL-6) levels done (samples taken on days one and five) were reported to be 10 *μ*g/ml (increase) and 2 *μ*/ml (normal), respectively (report received 12 days later).

On routine follow up the patient had no signs of gynaecological or peritoneal malignancy which were earlier pointing towards a Meigs syndrome-like picture. 

## 3. Discussion

Odontogenic myxoma (OM) is a rare benign tumour arising from tooth forming mesenchyme [4, 5]. Primary anaesthetic concern is maintaining a patent and protected airway while allowing for unhindered surgical access. With airway being the main concern, patient is extubated only after full neuromuscular reversal and no active bleeding is present in the surgical site, which might compromise the airway.

In our patient, all of the above-mentioned precautions were taken care of. Myxomas present elsewhere are known to cause serous cavity effusions [6, 7]. Suspicion of Meigs syndrome [8, 9] was negated by a finding of a small hemorrhagic ovarian cyst without any myxoid component [10, 11]. Suspicion of pseudo-meigs syndrome [12] was made by raised CA-125, massive ascites, and pleural, and pericardial effusions with small hemorrhagic ovarian cyst. Negative pleural and ascitic fluid cytology for malignant cells, premenarchal age group, and spontaneous resolution of all the findings without any surgical or medical intervention went against the diagnosis of any pelvic or abdominal malignancy. Benign hemorrhagic ovarian cysts are not known to cause such massive serous cavities effusion.

 It was later concluded that the postoperative events were not due to any ovarian pathology, but due to mediators produced by the primary tumour itself. The incidental finding of raised CA-125 was due to hemorrhagic ovarian cyst as it decreased spontaneously. As IL-6 was raised in the sample taken during the event and subsided thereafter coinciding with relief of symptoms, it can be proposed that the primary tumour released factors responsible for clinical findings. IL-6 is known to cause capillary leakage syndrome and might be responsible along with other mediators for the hemodynamic collapse and serous cavity effusions in concert with fluid resuscitation [3].

In summary, we suggest that odontogenic myxofibroma patients be monitored postoperatively for occurrence of inflammatory mediator related complications apart from the usual airway complications.
